# UK population norms for the modified dental anxiety scale with percentile calculator: adult dental health survey 2009 results

**DOI:** 10.1186/1472-6831-13-29

**Published:** 2013-06-24

**Authors:** Gerry Humphris, John R Crawford, Kirsty Hill, Angela Gilbert, Ruth Freeman

**Affiliations:** 1 Health Psychology, University of St Andrews, Scotland, UK; 2 School of Psychology, University of Aberdeen, Scotland, UK; 3 Dental Public Health, University of Birmingham, England, UK; 4 Dundee Dental School, University of Dundee, Scotland, UK; 5 DHSRU, University of Dundee, Scotland, UK; 6 Dental Public Health, NHS Tayside, Scotland, UK

**Keywords:** Dental anxiety, Representative survey, Psychometrics, Percentiles, On-line calculator

## Abstract

**Background:**

A recent UK population survey of oral health included questions to assess dental anxiety to provide mean and prevalence estimates of this important psychological construct.

**Methods:**

A two-stage cluster sample was used for the survey across England, Wales, and Northern Ireland. The survey took place between October-December 2009, and January-April 2010. All interviewers were trained on survey procedures. Within the 7,233 households sampled there were 13,509 adults who were asked to participate in the survey and 11,382 participated (84%).

**Results:**

The scale was reliable and showed some evidence of unidimensionality. Estimated proportion of participants with high dental anxiety (cut-off score = 19) was 11.6%. Percentiles and confidence intervals were presented and can be estimated for individual patients across various age ranges and gender using an on-line tool.

**Conclusions:**

The largest reported data set on the MDAS from a representative UK sample was presented. The scale’s psychometrics is supportive for the routine assessment of patient dental anxiety to compare against a number of major demographic groups categorised by age and sex. Practitioners within the UK have a resource to estimate the rarity of a particular patient’s level of dental anxiety, with confidence intervals, when using the on-line percentile calculator.

## Background

In 2009 the fifth decennial national survey of the adult dental health in the United Kingdom was conducted. The first national UK survey took place in 1968. In the earlier surveys dental anxiety was assessed using single item questions [1,2] which did not allow reliable measurement. RF and KH were involved in the development of the question bank for the 2009 survey. The Modified Dental Anxiety Scale (MDAS) was included as it had been shown to be a reliable and valid instrument within [3] and between countries [4]. This paper takes the opportunity to report on this aspect of the Adult Dental Health Survey (ADHS), 2009 to provide population norms and percentiles for use by dentists.

The Modified Dental Anxiety Scale is a brief, 5 item questionnaire with a consistent answering scheme for each item ranging from ‘not anxious’ to ‘extremely anxious’ [3]. It is summed together to construct a Likert scale with a minimum score of 5 and a maximum of 25. It is the most frequently used dental anxiety questionnaire in the UK [5] and does not increase patient fears when completed [6,7]. Existing data suggest that completion of the questionnaire can significantly reduce state anxiety in the practice setting [8,9]. It has defendable psychometric properties, is relatively quick and simple to complete and score [4,10]. A cut-off value of 19 and above has been determined empirically [3,11] to recommend to dental professionals that possible additional assistance may be required for successful treatment completion. The measure has been used in numerous research [12-14] and clinical-related [12,15] studies and contributed to our knowledge of this important psychological construct. It is one of a number of instruments that have been designed to help study the properties of dental anxiety [16]. The scale is based on the original Corah’s Dental Anxiety Scale (CDAS) [17] for which conversion tables have been published to compare values between the two instruments [18]. The MDAS has been translated into a number of languages, many of which have published psychometrics (Spanish [19], Turkish [20], Greek [21], Chinese [22], Tamil [23] and Arabic [24]).

Data from UK samples are available [10,12,14,25] to allow clinicians to compare the scores of their patients. However, these samples may not have been representative. From this position, the key advisors (RF and KH) on barriers to dental health, made recommendations to the organisational team of the 2009 Adult Dental Health Survey to include the MDAS as a tool for assessing dental anxiety in the UK [9]. The new data would report an important outcome, namely: the unrivalled set of UK norms and provide a valuable comparator for patient assessments. Further, researchers or clinicians may benefit from access to percentiles tabulated across major demographic groups to enhance this comparison. The advantage of percentiles is that they can identify the rarity of a patient’s score, and hence provide information supplementary to simply being above or below a cut-off [26].

A second outcome of the normative component of the study was to provide *interval* estimates of the percentile ranks corresponding to raw scores on the MDAS. When health professionals refer a patient’s score to percentile norms, their interest is in the standing (percentile rank) of the patient’s score in the normative *population*, rather than its standing in the particular group of participants who happen to make up the normative *sample.* Although, in the present case, the normative sample used to provide the basis of conversion from raw scores to percentile ranks was very large, it is still the case that, as with any normative data, there is some uncertainty about these quantities. Thus the percentile rank for a raw score obtained from a normative sample must be viewed as a point estimate of the percentile rank of the score in the population and should be accompanied by an interval estimate [26]. Interval estimates serve the useful general purpose of reminding us that all normative data are fallible and serve the specific purpose of quantifying this fallibility [27,28].

Hence, the aims of this paper were threefold: first to evaluate the psychometric properties of the MDAS in a large representative sample. Second, to report a set of norms (in the form of percentile ranks) for the adult UK population, thereby providing clinicians with reference values for their patient’s scores. Third, to supplement the *point* estimates of the percentile ranks with *interval* estimates.

## Methods

### Sample and procedure

A two-stage cluster sample was used for the survey comprising of 253 primary sampling units (PSU) across England and Wales, and a further 15 PSUs in Northern Ireland. Each PSU consisted of two postcode sectors with 25 addresses sampled from each, giving a total sample of 13,400 addresses. Of these 12,054 were eligible for inclusion (1,346 ineligibles were unoccupied households, business addresses, care homes etc.). These procedures were consistent with previous ADHS collections using multi-stage stratified sampling. Postcode sectors were paired together to help reduce the effects of clustering and increase the diversity of the population within each PSU. The pairing of neighbouring postcode sectors also helped reduce the design effect [13]. The standard approach in the Office of National Statistics is to pair off contiguous PSUs into collapsed strata, and to base the variance of the estimator on the squared differences between PSUs within strata, summed over strata. The ONS policy is that it would not be appropriate to mix the postcode sector PSUs from multistage samples with those of single stage samples of households, therefore PSUs in the ADHS are paired [29]. In each of the 10 English Strategic Health Authorities and in Wales, 1,150 addresses were sampled and 750 addresses were sampled in Northern Ireland. The survey took place between October-December 2009, and January-April 2010. All interviewers were trained on survey procedures.

Of the 12,054 eligible households, 7,233 participated (60% household response rate), while the remaining 3,895 households refused to participate or were non-contactable (n = 455) or other non-response (n = 471). Within the 7,233 households there were 13,509 adults who were asked to participate in the survey - of these 11,382 participated (84%). All individuals aged 16 yearsand older were invited to participate. So we had a household response rate of 60% (7,233 HHs) and an individual response rate (from within those households) of 84% (11,382 individuals).

A two stage weighting approach was adopted which ensured that the 1,150 addresses were sampled in each English SHA and in Wales, and a further 750 in Northern Ireland. A consequence of the aim to achieve similar sample size samples at the SHA level is that differential sampling rates were utilized in the SHAs, Wales and Northern Ireland. A survey weight had to be employed to compensate for these differential rates. As well as this weighting to address the sample design deficiencies, weighting was also employed to reduce bias attributed to non-response. Unfortunately, minimal information is available about non-responding households: however geographic information associated with non-responding households is available from the 2001 Census. This Census categorises each PSU based upon key characteristics including typical household type, social-economic status, typical ethnicity etc. Hence household non-response was based on the area a household was in. Details of this are contained in the Technical Report [13].

### Questionnaire and measures

The ADHS included a clinical examination and a questionnaire [13]. The content included: major indicators of oral health and function, dental diseases, urgent conditions such as pain and sepsis, complex treatments received, oral health risk factors and behaviour, service considerations and outcomes including access and barriers to care. The breadth of subject areas is too great (p21 Foundation Report) in scope for inclusion in this paper. Hence it is the later aspect of barriers and specifically, dental anxiety, that this paper is focused. Regularity of attendance was established from the questionnaire. The wording was: “In general do you go to the dentist for… (1) a regular check up, (2) an occasional check up, (3) or only when you’re having trouble with your teeth/dentures”.

To assess dental anxiety we employed the MDAS, which asks participants to rate: how anxious one feels the day before a dental appointment, then when in the waiting room, waiting for the receipt of drilling, scaling and a local anaesthetic injection. Responses range from ‘not anxious’ (scored 1) to ‘extremely anxious’ (scored 5). The five items are summed to create a total score, which has a range from a minimum of 5 to a maximum of 25. Total scores of 5 and 25 would denote: no dental anxiety and extreme dental anxiety, respectively. Reliability of the English language version from original investigation [3] of the MDAS is good (internal consistency= 0.89; test-retest= 0.82). The scale can be downloaded:

http://medicine.st-andrews.ac.uk/supplemental/humphris/dentalAnxiety.htm. The item wording is reproduced in Table 1 and the scale layout can be reproduced from the dedicated website download.

**Table 1:** Psychometric details: item means (sd); factor loadings, maximum likelihood (ML), asymptotic distribution free (ADF), pearson correlation matrix and item wording

***Items***	***Mean***	***Sd***	***Factor loadings***		
			***ML***	***ADF***	
Q1	1.91	1.22	.94	.93	
Q2	1.98	1.26	.97	.97	
Q3	2.61	1.42	.79	.79	
Q4	1.69	1.09	.67	.67	
Q5	2.55	1.43	.66	.66	
***Items***	***Correlations***				
	***Q1***	***Q2***	***Q3***	***Q4***	***Q5***
Q1	1	.91	.74	.64	.62
Q2		1	.77	.65	.65
Q3			1	.62	.76
Q4				1	.55
Q5					1
***Items***	***Wording***				
Q1	If you went to the dentist for TREATMENT TOMORROW, how would you feel?				
Q2	If you were sitting in the WAITING ROOM (waiting for treatment), how would you feel?				
Q3	If you were about to have a TOOTH DRILLED, how would you feel?				
Q4	If you were about to have your TEETH SCALED AND POLISHED, how would you feel?				
Q5	If you were about to have a LOCAL ANAESTHETIC INJECTION in your gum above an upper back tooth, how would you feel?				

Cronbach’s alpha (95% CIs): 0.917 (0.915, 0.919).

Confirmatory Factor Analysis ‘closeness of fit’ estimates for unidimensional scale (ADF estimation):

chi-square = 19.28.

degrees of freedom = 2.

CFI = 0.998, (greater than 0.95).

TLI = 0.990, (greater than 0.95).

RMSEA (95% CIs) = 0.028 (0.017, 0.041) (less than 0.05).

### Procedure

The fieldwork procedures may be summarised as follows: the household was contacted initially by letter in advance of a household call. The household was informed that an interviewer would call to discuss the interview within a short period (days). To minimize the number of non-contacts (householders not contactable), all the interviewers were instructed to call at the addresses on different days, and at different times of the day (p17 Foundation Report) [13]. Participants were asked about demographic status, and other dental-related issues including the 5 questions of the MDAS by trained interviewers in the household.

### Ethical issues

A single application was submitted to NHS Research Ethics System (NRES) covering all aspects of the survey in England, Wales and Northern Ireland. Approval was granted in June 2009. All participants gave written consent.

### Statistical analysis

Data were analysed using SPSS™ version 19 and AMOS™ version 19 [30]. Internal consistency and confirmatory factor analysis (CFA) was performed to assess, respectively, the internal consistency (Cronbach’s alpha) and the level of fit (Chi-square, CFI, TLI & RMSEA) to a unidimensional model of scaling to a continuous latent construct. CFA was estimated using maximum likelihood and distribution free methods for comparison. All five items were described by a single latent variable. The first item was selected to set the factor coefficient to unity for identification purposes. This selection is generally regarded as arbitrary. Frequencies, means and standard deviations were calculated across the major demographic factors and self-reported visiting. A comparison was made between the original data set reported in 2008 and the current data using fixed factor analysis of variance, with and without adjustment for major demographic variables, namely: age, sex and socio-economic status.

A set of percentiles was prepared across gender and major age groups. A threshold of 19 and above was adopted [3,11], as the level for which is it likely that a dental practitioner would consider using additional approaches to manage the patient such as relaxation, systematic desensitisation or pharmacological adjunct. Fixed factor analysis of variance was performed utilizing the continuous scale data to inspect variation of dental anxiety across major demographic, behavioural and socioeconomic status factors. Significance level was set at the conventional 5%, two-tailed.

### Point estimates of percentile ranks

The standard method of obtaining percentile ranks was used [31,32]. That is,

(1) $$\mathrm{Percentile}\;\mathrm{Rank}=\left(\frac{m+0.5k}{N}\right)100,$$

where *m* is the number of members of the normative sample obtaining a score lower than the score of interest, *k* is the number obtaining the score of interest, and *N* is the overall normative sample size.

### Interval estimates of percentile ranks

As noted, a further aim of the present study was to accompany the *point* estimates of the percentile ranks corresponding to raw score with *interval* estimates of these quantities. A percentile rank is simply a proportion multiplied by 100 thus methods of obtaining an interval estimate of a proportion (such as classical methods based on the binomial distribution) can be used to obtain interval estimates of a percentile rank. However, for the present problem there is a complication. Although anxiety scores are discrete (i.e., integer-valued), the underlying dimension they index are generally taken to be continuous, real-valued quantities. Thus, a raw score of, say, 7 is regarded as a point estimate of a real-valued score which could lie anywhere in the interval 6.5 to 7.4999 (plus an infinite number of additional 9s after the 4^th^ decimal place). Put another way, in principle we could distinguish among individuals obtaining the same raw score were we to introduce tie-breaking items. This assumption of a continuous underlying score is ubiquitous in psychological measurement and motivates the standard definition of a percentile rank (formula 1).

Normative data for scales such as the MDAS will always contain a sizeable number of tied scores; that is, a large number of people in the normative sample will obtain the same raw test score. Indeed, if a normative sample is large and the data are skewed (as would usually be the case for anxiety scales as the majority of the general population are not clinically anxious), then there could literally be hundreds of such ties for a given raw score. The present problem therefore differs from those dealt with by standard binomial sampling in which there can be no possibility of multiple ties.

Crawford, Garthwaite and Slick [31] have recently developed Bayesian and classical methods that incorporate the additional uncertainty arising from tied scores. Crawford et al. [26] and Crawford et al. [33] have used these methods to provide interval estimates for self-report mood scales, such as the HADS, DASS, and PANAS; the methods have also been used to provide interval estimates for a variety of neuropsychological test scores [34,35]. In the present study we apply the Bayesian method to MDAS scores.

To illustrate the issue the methods address: suppose that in a normative sample of 100 people, 89 obtained lower scores than a case and 2 obtained the same score as the case. Then the point estimate of the percentile rank for the case’s score (using formula 1) is 90 and applying Crawford et al’s. [36] Bayesian method, the interval estimate is from 82.15 to 95.27. Suppose, however, that 85 obtained lower scores and 10 obtained the same score. The point estimate of the percentile rank is the same as in the foregoing example (90) but the interval estimate is from 79.79 to 97.10; the latter interval is wider because of the increased uncertainty introduced by the larger number of ties (10 versus 2). The technical details of these methods are not set out here: see Crawford et al. [36] for their derivation, and for an additional mathematical treatment and evaluation, see Garthwaite and Crawford [37].

### One-sided versus two-sided intervals

In practice there will be occasions in which a one-sided interval may be preferred over a two-sided interval. For example, a clinician may be interested in whether a patient’s score is less extreme than is indicated by the point estimate but not particularly interested in whether the score is even more extreme, or vice-versa. The methods developed by Crawford et al. [31] are easily adapted to provide a one-sided limit. However, without prior knowledge of which limit is of interest (the situation here, as the aim is to provide intervals for use by others) it is more convenient to generate 100(1-[*α*/2]) two-sided intervals which then provide 100(1-*α*) one-sided lower and upper limits. For example, if a 95% lower limit on the percentile rank is required then a 90% two-sided interval is generated: The user then simply disregards the upper limit of the two-sided interval and treats the lower limit as the desired one-sided 95% limit.

### Computer program for obtaining point and interval estimates of percentile ranks for raw scores on the MDAS

The point and interval estimates of percentile ranks for MDAS scores can be obtained using the tabled values provided in the present paper. However, we considered that some health professionals might find it more convenient if theses norms were also available via a computer program.

## Results

### Scale psychometrics

The psychometrics for the sample are provided in Table 1 including the means and standard deviations for each question response, internal consistency coefficient (Cronbach’s alpha) with 95% confidence intervals, and the results of confirmatory factor analysis (CFA) which were inspected for the unidimensional solution. Indices of fit include chi-square, CFI, TLI and RMSEA. Factor weightings between the latent variable and individual items are displayed including maximum-likelihood, and asymptotic distribution free estimates for comparison purposes. Note that 3 error covariances were specified. The internal consistency coefficient of 0.917 is regarded as ‘excellent’ [38]. The sample size of this study is sufficient to show narrow confidence intervals. The scale can be considered to be unidimensional as shown by the CFA and the indices of fit lying well within conventional limits of acceptability [39]. This was reflected in the near identical factor loadings obtained from maximum likelihood or distribution free estimation methods.

### Variation of dental anxiety

The total MDAS scores were found to vary significantly by the independent factors of gender, age, self-reported regularity of dental attendance and social status (Table 2). Females showed greater dental anxiety than men (F = 533.18, df = 1, 10084, *p* = 0.0001), a decreasing level with age category (overall F = 53.86, df = 6, 10079, *p* = 0.0001, linear effect: F = 293.35, *p* = 0.0001, quadratic effect: F = 18.01, *p* = .0001), increase with less regular visiting (F = 244.26, df = 2, 10083, *p* = 0.0001) and a weaker but significant effect of social status (F = 15.64, df = 6, 10079, *p* = 0.0001). On exclusion of the mixed category: the long term unemployed and unclassified, in the social status classification, the effect of social status on dental anxiety was linear (F = 25.12, *p* < .0001) and the degree of non-linearity was borderline non-significant (F = 2.40, *p* = 0.065). Prior to the exclusion of this mixed category the *p* levels, for the linearity testing, were 0.15 and .001 respectively.

**Table 2:** Frequency breakdown and N size for participant sample including MDAS means (SD), percent ≥ 19 score and proportion of variance explained by demographic and behavioural variables (eta-squared)

	**N**	**%**	**Mean**	**SD**	**% ≥19**	**eta**^**2**^
**Total**	**10086**	**88.7**	**10.65**	**5.55**	**12.1**	
*Sex*						
Male	4736	47.0	9.33	4.87	6.9	0.050
Female	5350	53.0	11.82	5.84	16.7	
*Age (years)*						
16-24	879	8.70	11.76	5.58	15.7	0.031
25-34	1317	13.1	11.72	5.62	16.0	
35-44	1793	17.8	11.30	5.70	14.5	
45-54	1794	17.8	10.98	5.66	13.5	
55-64	1796	17.8	10.52	5.66	12.0	
65-74	1490	14.0	9.19	4.81	6.3	
75 years and over	1097	10.9	8.97	4.86	6.1	
*Visiting the dentist*						
Regular	6413	63.6	9.81	4.87	7.7	0.046
Occasional check up	822	8.1	10.78	5.34	11.8	
When in pain/or trouble	2851	28.3	12.50	6.48	22.1	
*Occupation: SES*						
Managerial and professional occupations	3956	39.2	10.35	5.16	9.9	0.004
Intermediate occupations	816	8.1	10.94	5.61	12.5	
Small employers & own account workers	1119	11.1	10.43	5.48	11.0	
Lower supervisory and technical occupations	1117	11.1	10.90	5.78	14.1	
Semi-routine and routine occupations	2503	24.8	11.06	5.94	14.8	
Long term unemployed/ never worked	399	4.0	10.09	5.67	12.0	
Not classified	176	1.7	11.33	5.98	17.6	

### Obtaining point and interval estimates of the percentile ranks for raw scores

The percentile ranks for MDAS raw scores can be obtained using Tables 3, 4, 5, 6, 7, 8. As gender and age both influenced MDAS scores appreciably, the normative data were stratified by both these variables; three age bands were created (16 to 34, 35–54, 55+). The age bands were chosen as they conformed to the 3 bands reported in the ADHS report. In addition to providing the point estimates of the percentile ranks, the tables also provide 95% interval estimates. An expanded age breakdown (4 or more bands) was not selected as the percentile confidence intervals would become too large for practical interpretation. The overall mean levels of dental anxiety in the current survey (10.65, SD 5.55) and the previous 2008 survey report [25] (10.39, SD 5.46) was not significantly different (*p* = 0.16). This was demonstrated further when these means were adjusted for gender, age bands and social status classification (current study: mean = 10.61, 95% CIs 10.50, 10.73; 2008 report: mean = 10.50, 95% CIs 10.11, 10.89; F = 0.31, df (35, 10245), p = 0.58).

**Table 3:** Percentile ranks (point and 95% interval estimates) corresponding to MDAS raw scores for females aged 16 to 34

		**95% CI**	
**Raw score**	**Percentile rank**	**Lower**	**Upper**
5	5	0.2	9.8
6	12	9.1	15.7
7	18	14.4	22.5
8	25	20.9	28.5
9	31	26.7	36.0
10	37	33.9	41.2
11	43	38.8	47.2
12	49	45.0	53.8
13	55	51.4	58.9
14	60	56.5	64.4
15	66	62.1	69.5
16	70	67.1	73.6
17	74	71.1	77.2
18	78	74.7	80.3
19	81	78.1	84.4
20	85	82.6	87.8
21	89	86.1	91.2
22	92	89.7	93.7
23	94	92.4	95.8
24	96	94.6	97.1
25	98	96.3	99.9

**Table 4:** Percentile ranks (point and 95% interval estimates) corresponding to MDAS raw scores for females 35 to 54

		**95% CI**	
**Raw score**	**Percentile rank**	**Lower**	**Upper**
5	6	0.3	12.8
6	16	12.6	19.7
7	23	18.9	28.0
8	32	27.2	36.0
9	39	34.8	42.7
10	46	41.4	49.7
11	52	48.2	55.3
12	57	53.6	60.2
13	61	58.4	64.5
14	66	62.6	68.5
15	69	66.7	72.1
16	73	70.3	75.9
17	76	74.1	78.8
18	80	77.1	82.0
19	83	80.5	85.2
20	86	83.9	88.5
21	89	87.3	91.0
22	91	89.8	92.9
23	93	91.8	94.6
24	95	93.5	96.0
25	98	95.4	99.9

**Table 5:** Percentile ranks (point and 95% interval estimates) corresponding to MDAS raw scores for females 55 plus

		**95% CI**	
**Raw score**	**Percentile rank**	**Lower**	**Upper**
5	11	0.5	20.8
6	24	20.7	28.2
7	32	27.3	37.6
8	40	36.6	43.8
9	46	42.5	50.0
10	53	48.7	57.0
11	59	55.7	62.3
12	64	60.8	67.7
13	69	66.2	72.3
14	73	70.7	75.8
15	77	74.2	79.6
16	81	78.2	82.7
17	83	81.3	85.5
18	86	84.1	87.9
19	88	86.5	89.8
20	90	88.5	92.0
21	92	90.9	93.9
22	94	92.8	95.1
23	95	94.0	96.4
24	96	95.5	97.2
25	98	96.6	99.9

**Table 6:** Percentile ranks (point and 95% interval estimates) corresponding to MDAS raw scores for males 16 to 34

		**95% CI**	
**Raw score**	**Percentile rank**	**Lower**	**Upper**
5	9	0.4	19.0
6	23	18.3	28.2
7	32	26.5	38.3
8	42	36.4	47.1
9	49	44.7	54.3
10	56	51.6	60.7
11	62	57.9	66.5
12	68	63.7	71.7
13	73	69.1	77.2
14	78	74.7	81.5
15	82	78.8	84.7
16	85	82.2	87.9
17	88	85.4	89.9
18	90	87.4	91.6
19	92	89.3	93.5
20	94	91.6	95.3
21	95	93.7	96.9
22	97	95.4	97.8
23	98	96.4	98.6
24	98	97.4	99.1
25	99	98.2	100.0

**Table 7:** Percentile ranks (point and 95% interval estimates) corresponding to MDAS raw scores for males 35 to 54

		**95% CI**	
**Raw score**	**Percentile rank**	**Lower**	**Upper**
5	12	0.6	24.1
6	29	23.9	34.2
7	39	33.1	44.4
8	47	43.1	51.8
9	54	50.1	58.5
10	61	56.7	64.5
11	67	62.7	70.4
12	72	68.5	74.8
13	76	72.9	79.0
14	80	77.1	82.2
15	83	80.4	85.7
16	86	83.9	87.8
17	88	86.1	90.3
18	90	88.7	91.9
19	92	90.3	93.5
20	94	92.1	95.2
21	95	94.0	96.4
22	96	95.2	97.4
23	97	96.3	98.0
24	98	96.9	98.4
25	99	97.7	100.0

**Table 8:** Percentile ranks (point and 95% interval estimates) corresponding to MDAS raw scores for males 55 plus

		**95% CI**	
**Raw score**	**Percentile rank**	**Lower**	**Upper**
5	18	0.9	36.2
6	42	36.6	48.1
7	53	47.2	58.4
8	61	57.3	65.4
9	68	64.1	72.2
10	75	71.0	78.3
11	80	76.9	82.5
12	84	81.1	86.3
13	87	84.9	88.8
14	89	87.3	90.6
15	91	89.2	92.6
16	93	91.3	94.0
17	94	92.7	95.2
18	95	94.1	96.3
19	96	95.1	96.9
20	97	95.8	97.6
21	98	96.6	98.2
22	98	97.4	98.6
23	98	97.8	98.9
24	99	98.1	99.2
25	99	98.6	100.0

### Computer program for scoring the MDAS

As previously noted, a compiled (i.e. ready-to-run) computer program for PCs, MDAS_PRs.exe, was written (using the Delphi programming language) to express a patient’s raw score on the MDAS as a percentile rank with accompanying interval estimate (the program will also run on a Mac provided that suitable PC emulation software is installed). The program is free and can be downloaded (either as an uncompressed executable or as a zip file) from the first author’s web pages at http://medicine.st-andrews.ac.uk/supplemental/humphris/dentalAnxiety.htm, or from http://homepages.abdn.ac.uk/j.crawford/pages/dept/MDAS_PRs.htm (After downloading the program it can be run by clicking on the program in windows explorer, or, if a shortcut to the program has been created on the user’s desktop, by clicking on the shortcut icon).

The program prompts the user to select the patient’s gender and age using radio buttons and to enter their MDAS raw score. The output from the program consists of the patient’s raw score on the MDAS (as entered by the user), and the point and interval estimates of the percentile rank for the score. These results can be viewed on screen, saved to a file, and/or printed.

## Discussion

The psychometric data appear to show that the MDAS is a reliable unidimensional scale. The total score therefore can be interpreted as a reasonable function of the latent construct of dental anxiety. The fit statistics for the confirmatory factor analysis are favourable as shown, for example, by the upper value of the 95% confidence interval of the RMSEA being less than 0.05 [15]. The 3 correlated errors between the last 3 items of the scale were required to achieve this ‘close’ fit which was not regarded as unreasonable, but can indicate an element of minor ‘strain’ in the uni-dimensional model preferred for the calculation of a clinical useful scale. Assumptions about normality of scale items appeared to be justified by the almost identical parameter estimates of factor score loadings computed via maximum likelihood or distribution free methods. This analysis provides reassurance that the promotion of the items to interval measurement assumptions did not deviate from the pattern of factor coefficients compared with a less constrained distributional item profile.

The association of dental anxiety with gender was highly significant. This dichotomous variable explained 5% of the variance of dental anxiety assessed by the MDAS. Previous literature is consistent with the direction of this relationship [40,41]. Similarly the weakening level of dental anxiety with increasing age has been repeatedly reported in community samples from developed countries [3,25]. This negative relationship with age in years can be explained powerfully using linear and curvi-linear equations. Examination of the reduction of dental anxiety shows a significant decline in dental anxiety beyond the age of 54 years (a drop of almost 0.7 of a unit score). In addition, the two age groups within the range of 16–34 years were found to report nearly identical mean levels of dental anxiety (11.76 and 11.72 respectively), reflecting a stable mean level of dental anxiety. Hence, the sample was split into three principle age groups for the benefit of calculating percentiles, namely: 16–34, 35–54 and 55+ years. The effect of the association with self-reported regularity of dental visiting and dental anxiety was in the expected direction and closely matches previous research findings [42,43]. The relationship between dental anxiety and social status was linear, as has been previously shown [44] but this was apparent only when the mixed category of ‘long term unemployed and never worked’ had been removed from linearity testing.

The large scale normative data provided here, and via the accompanying computer program, provide clinicians with a simple means of quantifying the extremity or otherwise of a patient’s level of dental anxiety. For example, suppose that a patient is male, is 60 years of age, and obtains a raw score of 22 on the MDAS. Then from Table 8 (or via the computer program), it can be seen that his score is at the 98th percentile. That is, 98% of individuals in the normative population are expected to obtain a lower score than the patient’s score. It can also be seen that there is very little uncertainty over the aforementioned point estimate. The 95% interval estimate ranges from 97.4 to 98.6.

It is generally the case that, for moderate-to-high MDAS scores, the interval estimates of the percentile ranks for MDAS scores will be fairly narrow, thereby indicating that the point estimates obtained using the MDAS normative sample provides an accurate estimate of the true percentile rank of the raw score in the population. However, there will be considerably more uncertainty attached to percentile ranks for low scores. This occurs because a large number of the normative sample obtain low scores (that is, there is a large number of ties as many members of the general adult population do not suffer from dental anxiety) and thus a higher degree of uncertainty over an individual’s percentile rank. It will be appreciated however, that this is not of much practical concern as there is little need for a precise estimate for low scores.

### Bayesian versus classical interpretations of the interval estimate on a score’s percentile rank

The method used in the present paper to provide interval estimates of a patient’s percentile ranks is a Bayesian method. As Antelman [45] notes, the frequentist (classical) conception of a confidence interval is that, “It is one interval generated by a procedure that will give correct intervals 95% of the time. Whether or not the one (and only) interval you happened to get is correct or not is unknown” (p. 375). Thus, in the present context, the classical interpretation of the interval estimate on the percentile rank for a raw score on the MDAS is as follows, “if we could compute a confidence interval for each of a large number of normative samples collected in the same way as the present MDAS normative sample, about 95% of these intervals would contain the true percentile rank of the patient’s score”.

The Bayesian interpretation of such an interval is “there is a 95% probability that the true percentile rank of the patient’s score lies within the stated interval”. This statement is not only less convoluted but it also captures what a clinician would wish to conclude from an interval estimate [46]. Indeed most health professionals who use classical/frequentist confidence limits probably construe these in what are essentially Bayesian terms [47].

Finally, for the present problem the Bayesian approach used here exhibits a very high degree of convergence with its classical equivalents [31]. For example, applying Crawford et al’s. [31] classical Clopper-Pearson mid-*p* method to the examples featured earlier yielded intervals that were identical to the Bayesian interval to two decimal places. This convergence between different methods is reassuring regardless of whether a health professional is Bayesian, frequentist or eclectic in orientation.

### Confidence intervals capturing sampling error versus measurement error

The confidence intervals on the percentile ranks provided here should not be confused with confidence limits derived from classical *test* theory that attempt to capture the effects of measurement error on an individual’s score [48]. When the latter intervals are used, the clinician is posing the question “assuming scores are normally distributed, and assuming no error in estimating the population mean, standard deviation and reliability coefficient of the test, how much uncertainty is there over an individual’s score as a function of measurement error in the scale?” [49,50]. In contrast, when using the intervals presented in the present paper, the concern is solely with the score *in hand*. The more concrete question posed is “how much uncertainty is there over the standing (i.e., percentile rank) of the patient’s MDAS score as a function of error in using a normative sample to estimate its standing in the normative population. That is, they do not address the issue of what score an individual might obtain on another occasion, or on a set of alternative, parallel items, but simply provide interval estimates for the percentage of the normative population who would score below the score obtained by the individual [26].

### Limitations

We have assumed that the individual reports of dental anxiety are independent of each other regardless of whether there was more than one participant in each household. Furthermore, all surveys have deficiencies in sampling that have been addressed as far as possible by the statistical team that ran the technical report for the survey. Representativeness may be considered, at the very least, partially achieved, for example,we compared the proportions of the ADHS sample with the Census 2007 for the UK for the comparable age groups 25–54 years, 55–64 years and 65 years and over. The differences in proportions were 0.05, 0.02 and 0.03 respectively which confirmed that the ADHS sample was a close approximation to the UK population. The authors are relatively relaxed with making the assumption of generalizability, but we do accept there are minor imperfections. However, the survey has been endorsed by policy makers and public health planners as an important addition to obtaining a picture of the dental health, attitudes and behaviour within the UK, including estimates of dental anxiety.

## Conclusions

The tabled values provided here, and the accompanying computer program, provide a quick and reliable means of obtaining percentile norms for the MDAS. The percentile norms allow health professionals to quantify the rarity, or otherwise, of the patients’ scores. Finally, the provision of interval estimates for the percentile rank of a score serves the general purpose of reminding clinicians that all normative data are fallible. It also serves the specific and practical purpose of quantifying the uncertainty over the standing of an individual’s score when referred to such data.

## Competing interests

The authors declare that they have no competing interests.

## Authors’ contributions

RF and KH were part of the research team for the Adult Dental Health Survey for the UK, 2009. GH, KH, AG & RF conceived the study. GH & JC ran the psychometric and Bayesian analyses respectively. JC constructed the percentile calculator. All authors contributed to various drafts and agreed the contents. RF finalized the submitted manuscript. All authors read and approved the final manuscript.

## Pre-publication history

The pre-publication history for this paper can be accessed here:

http://www.biomedcentral.com/1472-6831/13/29/prepub
